# *N*-[2-(3,4-Di­meth­oxy­phen­yl)-2-(phenyl­sulfan­yl)eth­yl]-2-(2-fluoro­phen­yl)acetamide

**DOI:** 10.1107/S2414314620016636

**Published:** 2021-01-05

**Authors:** Marco A. Ovalle, José A. Romero, Gerardo Aguirre

**Affiliations:** a Tecnológico Nacional de México/Instituto Tecnológico de Tijuana, Centro de Graduados e Investigación en Química. Apartado Postal 1166, Tijuana, B.C., Mexico; University of Otago, New Zealand

**Keywords:** crystal structure, medicinal chemistry, anti­tuberculosis activity

## Abstract

The title compound, C_24_H_24_FNO_3_S, is an inter­mediate in the synthesis of fluorine containing iso­quinoline alkaloids, which crystallizes in the triclinic space group *P*  with one mol­ecule in the asymmetric unit. The structure presents a racemic mixture of enanti­omers. The C—S—C—C torsion angle between the benzene ring system and the sulfonyl benzene ring is −178.5 (1)°. In the crystal, N—H⋯O hydrogen bonds between neighbouring mol­ecules form chains of mol­ecules along the *a*-axis direction.

## Abstract 2

The title compound is an inter­mediate for the synthesis of iso­quinoline fluorine analogues. The structure presents a racemic mixture of enanti­omers. In the crystal, N—H⋯O hydrogen bonds between neighbouring mol­ecules form chains of mol­ecules along the *a*-axis direction.

## Graphical abstract

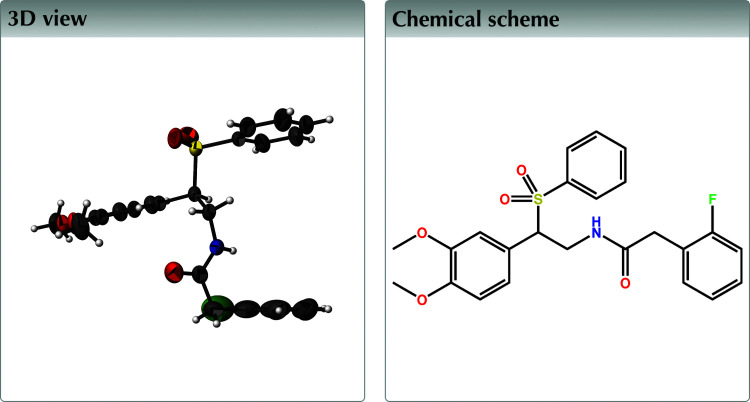

## Structure description

**Figure 1:**
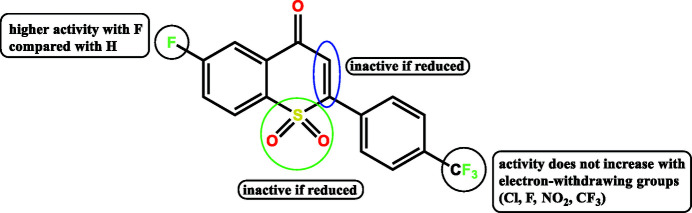
Structure–activity relationships of thio­chroman-4-one derivatives

Sulfur- and fluorine-containing mol­ecules play important roles in medicinal chemistry. Sulfur-containing compounds often show a variety of biological activities and serve important functions in applications in the pharmaceutical industry (Bernardi *et al.*, 1985 ▸). A variety of sulfur-containing mol­ecules have been isolated from natural sources and play major roles in drug discovery and development. The role of fluorine in drug design and development is expanding rapidly, as more is learned about the unique properties associated with this unusual element and how to deploy it in the pharmaceutical industry. The introduction of fluorine into a mol­ecule can influence conformation, p*K*_a_, intrinsic potency, membrane permeability, metabolic pathways, and pharmacokinetic properties (Gillis *et al.*, 2015 ▸). Various sulfur- and fluorine-containing mol­ecules have been studied for their applications in medicinal chemistry, with those containing a sulfone group emerging with promising results. Some examples are the recently reported thio­chroman-4-one derivatives (Vargas *et al.*, 2017 ▸), in which structure–activity relationships have been studied and it has been found that the vinyl sulfone and fluorine moieties play important roles in the biological activity of the mol­ecules (Fig. 1 ▸). The literature also reveals that these types of compounds also serve as neuroprotective agents (Woo *et al.*, 2014 ▸), exhibit anti­tuberculosis activity (Tiwari *et al.*, 2015 ▸) and inhibit prostate cancer (Goa & Spencer, 1998 ▸).

**Figure 2:**
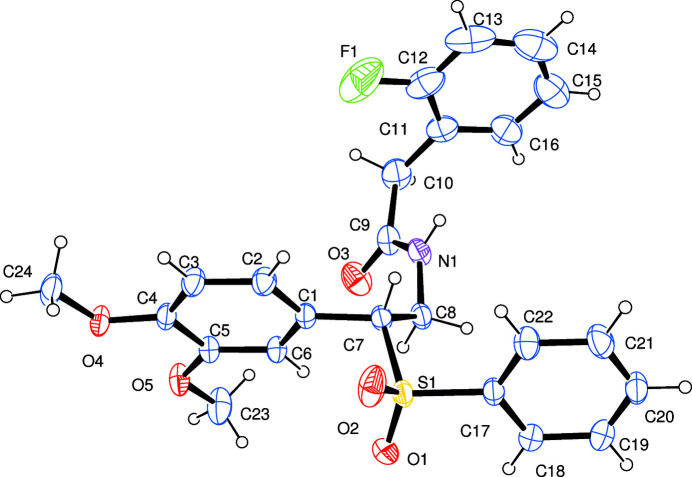
The mol­ecular structure of the title compound with displacement ellipsoids drawn at the 30% probability level.

**Figure 3:**
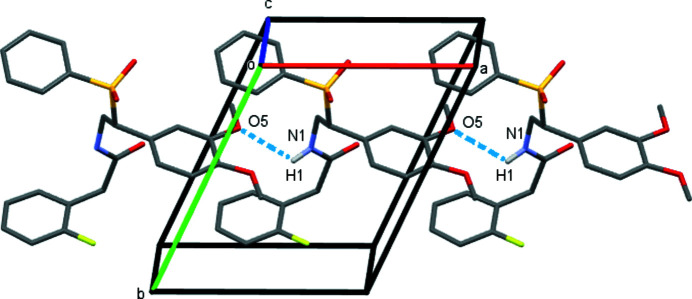
Crystal packing of the title compound viewed along the *c* axis. Inter­molecular hydrogen bonds are shown as dashed blue lines. Hydrogen atoms not involved in hydrogen bonding are omitted for clarity.

Continuing our inter­est in developing new sulfur- and fluorine-containing C17 S1 C7 C1biologically active alkaloids, we report here the synthesis and characterization of the title compound (Fig. 2 ▸) as a racemic mixture. The torsion angle between the benzene ring system and the sulfonyl benzene ring is −178.5 (1)°. The C11—C10—C9 angle [117.8 (2)°] is slightly widened in comparison to an *sp* ^3^-hybridized carbon atom; this is probably due to an attractive inter­action between the fluorine on the benzene ring and the hydrogen atoms on the benzyl carbon. In the crystal, N—H⋯O hydrogen bonds between neighbouring mol­ecules form chains of mol­ecules along the *a*-axis direction (Table 1 ▸; Fig. 3 ▸).

## Synthesis and crystallization

The title compound was synthesized by the oxidation of *N*-(2-(3,4-di­meth­oxy­phen­yl)-2-(phenyl­thio)­eth­yl)-2-(2-fluoro­phen­yl)acetamide (0.229 g, 0.54 mmol) treated with NaIO_4_ (0.264, 1.23 mmol) in water (6 ml). The reaction mixture was stirred for 2 h in reflux and then was allowed to cool down at room temperature prior to extractions with DCM (3 × 20 ml). The solvent in the combined organic layer was removed under vacuum and purified by flash chromatography on silica gel (DCM/MeOH 95:5) to give a pale-yellow solid in 67% yield (0.163 g, 0.36 mmol).

## Refinement

Crystal data, data collection and structure refinement details are summarized in Table 2 ▸.

## Supplementary Material

Crystal structure: contains datablock(s) I. DOI: 10.1107/S2414314620016636/sj4219sup1.cif

Structure factors: contains datablock(s) I. DOI: 10.1107/S2414314620016636/sj4219Isup2.hkl

Supporting information file. DOI: 10.1107/S2414314620016636/sj4219Isup3.cml

CCDC reference: 2052869

Additional supporting information:  crystallographic information; 3D view; checkCIF report

## Figures and Tables

**Table 1:** Hydrogen-bond geometry (Å, °)

*D*—H⋯*A*	*D*—H	H⋯*A*	*D*⋯*A*	*D*—H⋯*A*
N1—H1⋯O4^i^	0.86	2.46	3.207 (2)	145
N1—H1⋯O5^i^	0.86	2.50	2.945 (3)	113

Symmetry code: (i) 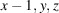 .

**Table 2:** Experimental details

Crystal data	
Chemical formula	C_24_H_24_FNO_5_S
*M*_r_	457.50
Crystal system, space group	Triclinic, *P* 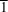
Temperature (K)	293
*a*, *b*, *c* (Å)	8.3751 (3), 9.7640 (4), 15.3592 (5)
α, β, γ (°)	97.676 (3), 93.885 (3), 115.319 (4)
*V* (Å^3^)	1114.18 (8)
*Z*	2
Radiation type	Cu *K*α
μ (mm^−1^)	1.68
Crystal size (mm)	0.21 × 0.12 × 0.07

Data collection	
Diffractometer	Rigaku SuperNova, Dual, Cu at zero, AtlasS2
Absorption correction	Multi-scan (*CrysAlis PRO*; Rigaku OD, 2015 ▸)
*T*_min_, *T*_max_	0.816, 1.000
No. of measured, independent and observed [*I* > 2σ(*I*)] reflections	14171, 4663, 4030
*R*_int_	0.022
(sin θ/λ)_max_ (Å^−1^)	0.631

Refinement	
*R*[*F* ^2^ > 2σ(*F* ^2^)], *wR*(*F* ^2^), *S*	0.052, 0.151, 1.08
No. of reflections	4663
No. of parameters	291
H-atom treatment	H-atom parameters constrained
Δρ_max_, Δρ_min_ (e Å^−3^)	0.37, −0.30

Computer programs: *CrysAlis PRO* (Rigaku OD, 2015 ▸), *SIR2004* (Burla *et al.*, 2007 ▸), *SHELXL* (Sheldrick, 2015 ▸), *Mercury* (Macrae *et al.*, 2020 ▸) and *publCIF* (Westrip, 2010 ▸).

## full crystallographic data

### Crystal data

**Table:**

C_24_H_24_FNO_5_S	*Z* = 2
*M**_r_* = 457.50	*F*(000) = 480
Triclinic, *P*1	*D*_x_ = 1.364 Mg m^−^^3^
*a* = 8.3751 (3) Å	Cu *K*α radiation, λ = 1.54184 Å
*b* = 9.7640 (4) Å	Cell parameters from 6250 reflections
*c* = 15.3592 (5) Å	θ = 2.9–76.6°
α = 97.676 (3)°	µ = 1.68 mm^−^^1^
β = 93.885 (3)°	*T* = 293 K
γ = 115.319 (4)°	Irregular, translucent intense colourless
*V* = 1114.18 (8) Å^3^	0.21 × 0.12 × 0.07 mm

### Data collection

**Table:**

Rigaku SuperNova, Dual, Cu at zero, AtlasS2 diffractometer	4663 independent reflections
Radiation source: micro-focus sealed X-ray tube, SuperNova (Cu) X-ray Source	4030 reflections with *I* > 2σ(*I*)
Mirror monochromator	*R*_int_ = 0.022
Detector resolution: 5.1980 pixels mm^-1^	θ_max_ = 76.8°, θ_min_ = 2.9°
ω scans	*h* = −10→10
Absorption correction: multi-scan (CrysAlisPro; Rigaku OD, 2015)	*k* = −12→12
*T*_min_ = 0.816, *T*_max_ = 1.000	*l* = −19→17
14171 measured reflections	

### Refinement

**Table:**

Refinement on *F*^2^	Primary atom site location: structure-invariant direct methods
Least-squares matrix: full	Hydrogen site location: inferred from neighbouring sites
*R*[*F*^2^ > 2σ(*F*^2^)] = 0.052	H-atom parameters constrained
*wR*(*F*^2^) = 0.151	*w* = 1/[σ^2^(*F*_o_^2^) + (0.0751*P*)^2^ + 0.4124*P*] where *P* = (*F*_o_^2^ + 2*F*_c_^2^)/3
*S* = 1.08	(Δ/σ)_max_ = 0.001
4663 reflections	Δρ_max_ = 0.37 e Å^−^^3^
291 parameters	Δρ_min_ = −0.30 e Å^−^^3^
0 restraints	

### Special details

**Table:**

Geometry. All esds (except the esd in the dihedral angle between two l.s. planes) are estimated using the full covariance matrix. The cell esds are taken into account individually in the estimation of esds in distances, angles and torsion angles; correlations between esds in cell parameters are only used when they are defined by crystal symmetry. An approximate (isotropic) treatment of cell esds is used for estimating esds involving l.s. planes.
Refinement. The C-bound H atoms were placed in calculated positions and treated as riding: C—H = 0.95–0.99 A° with *U*_iso_(H) = 1.5Ueq(Cmethyl) and 1.2Ueq(C) for other H atoms.

### Fractional atomic coordinates and isotropic or equivalent isotropic displacement parameters (Å^2^)

**Table:**

	*x*	*y*	*z*	*U*_iso_*/*U*_eq_	
S1	0.39652 (6)	0.22548 (6)	0.61360 (4)	0.04490 (17)	
F1	0.6743 (4)	0.9600 (3)	0.84275 (19)	0.1440 (11)	
O1	0.4440 (2)	0.1166 (2)	0.64885 (15)	0.0685 (5)	
O2	0.4379 (2)	0.2591 (2)	0.52804 (11)	0.0733 (6)	
O3	0.6887 (3)	0.5399 (3)	0.93514 (12)	0.0724 (5)	
O4	1.24722 (18)	0.61837 (19)	0.69344 (11)	0.0505 (4)	
O5	1.09028 (19)	0.4413 (2)	0.80129 (10)	0.0524 (4)	
N1	0.4765 (2)	0.5359 (2)	0.83493 (12)	0.0482 (4)	
H1	0.421805	0.586998	0.818120	0.058*	
C1	0.7007 (2)	0.4691 (2)	0.69245 (13)	0.0387 (4)	
C2	0.7860 (3)	0.5660 (3)	0.63531 (15)	0.0480 (5)	
H2	0.720985	0.596306	0.597378	0.058*	
C3	0.9689 (3)	0.6187 (3)	0.63396 (15)	0.0493 (5)	
H3	1.024558	0.683868	0.595053	0.059*	
C4	1.0682 (2)	0.5755 (2)	0.68960 (14)	0.0416 (4)	
C5	0.9826 (3)	0.4781 (2)	0.74848 (13)	0.0401 (4)	
C6	0.8009 (2)	0.4252 (2)	0.74868 (13)	0.0400 (4)	
H6	0.744532	0.359023	0.787008	0.048*	
C7	0.5010 (2)	0.4060 (2)	0.69177 (13)	0.0395 (4)	
H7	0.464425	0.478089	0.667997	0.047*	
C8	0.4351 (3)	0.3881 (3)	0.78194 (14)	0.0443 (5)	
H8A	0.307058	0.324890	0.773090	0.053*	
H8B	0.490585	0.336217	0.813264	0.053*	
C9	0.5952 (3)	0.5977 (3)	0.90877 (15)	0.0517 (5)	
C10	0.6108 (4)	0.7481 (3)	0.95980 (18)	0.0684 (7)	
H10A	0.599310	0.736563	1.021142	0.082*	
H10B	0.730226	0.827165	0.958892	0.082*	
C11	0.4797 (4)	0.8064 (3)	0.92863 (16)	0.0598 (6)	
C12	0.5128 (6)	0.9088 (4)	0.8713 (2)	0.0853 (10)	
C13	0.3893 (8)	0.9597 (4)	0.8421 (2)	0.1011 (14)	
H13	0.416268	1.030105	0.803685	0.121*	
C14	0.2253 (7)	0.9011 (5)	0.8724 (3)	0.1040 (13)	
H14	0.139973	0.931991	0.853658	0.125*	
C15	0.1876 (6)	0.8014 (5)	0.9281 (3)	0.0930 (11)	
H15	0.076739	0.762740	0.947768	0.112*	
C16	0.3134 (4)	0.7560 (4)	0.9562 (2)	0.0728 (8)	
H16	0.285373	0.687787	0.995874	0.087*	
C17	0.1652 (3)	0.1620 (2)	0.61424 (14)	0.0426 (4)	
C18	0.0725 (3)	0.0374 (3)	0.65450 (19)	0.0578 (6)	
H18	0.130805	−0.013477	0.679429	0.069*	
C19	−0.1083 (4)	−0.0101 (3)	0.6570 (2)	0.0741 (8)	
H19	−0.171943	−0.092894	0.684308	0.089*	
C20	−0.1935 (3)	0.0647 (3)	0.6194 (2)	0.0682 (7)	
H20	−0.315207	0.031227	0.620524	0.082*	
C21	−0.1021 (3)	0.1872 (4)	0.58042 (19)	0.0648 (7)	
H21	−0.161059	0.238211	0.556282	0.078*	
C22	0.0794 (3)	0.2369 (3)	0.57638 (16)	0.0552 (6)	
H22	0.141613	0.319261	0.548562	0.066*	
C23	1.0135 (3)	0.3553 (4)	0.86799 (19)	0.0692 (8)	
H23A	0.927690	0.253193	0.840542	0.104*	
H23B	0.955450	0.405134	0.902463	0.104*	
H23C	1.105425	0.349513	0.905972	0.104*	
C24	1.3384 (3)	0.7113 (3)	0.6318 (2)	0.0645 (7)	
H24A	1.287198	0.656772	0.572378	0.097*	
H24B	1.462304	0.733861	0.640809	0.097*	
H24C	1.326957	0.805723	0.640687	0.097*	

### Atomic displacement parameters (Å^2^)

**Table:**

	*U*^11^	*U*^22^	*U*^33^	*U*^12^	*U*^13^	*U*^23^
S1	0.0308 (2)	0.0492 (3)	0.0513 (3)	0.0168 (2)	0.00980 (19)	−0.0007 (2)
F1	0.155 (2)	0.1078 (18)	0.128 (2)	0.0098 (17)	0.0651 (18)	0.0350 (16)
O1	0.0445 (9)	0.0507 (9)	0.1138 (16)	0.0277 (8)	0.0078 (9)	0.0042 (9)
O2	0.0508 (10)	0.0919 (14)	0.0495 (10)	0.0093 (9)	0.0176 (8)	−0.0057 (9)
O3	0.0679 (12)	0.0981 (15)	0.0585 (11)	0.0487 (11)	−0.0068 (9)	0.0035 (10)
O4	0.0292 (7)	0.0595 (9)	0.0602 (9)	0.0132 (6)	0.0144 (6)	0.0205 (7)
O5	0.0329 (7)	0.0771 (11)	0.0533 (9)	0.0249 (7)	0.0114 (6)	0.0270 (8)
N1	0.0403 (9)	0.0568 (11)	0.0489 (10)	0.0267 (8)	0.0018 (7)	−0.0023 (8)
C1	0.0294 (9)	0.0432 (10)	0.0413 (10)	0.0145 (8)	0.0053 (7)	0.0057 (8)
C2	0.0393 (10)	0.0534 (12)	0.0534 (12)	0.0199 (9)	0.0061 (9)	0.0189 (10)
C3	0.0398 (11)	0.0536 (12)	0.0552 (12)	0.0164 (9)	0.0138 (9)	0.0239 (10)
C4	0.0288 (9)	0.0451 (10)	0.0465 (11)	0.0115 (8)	0.0105 (8)	0.0080 (8)
C5	0.0311 (9)	0.0493 (11)	0.0390 (10)	0.0166 (8)	0.0069 (7)	0.0088 (8)
C6	0.0316 (9)	0.0484 (11)	0.0404 (10)	0.0161 (8)	0.0112 (7)	0.0121 (8)
C7	0.0314 (9)	0.0442 (10)	0.0438 (10)	0.0180 (8)	0.0066 (7)	0.0062 (8)
C8	0.0333 (9)	0.0504 (11)	0.0499 (11)	0.0197 (8)	0.0105 (8)	0.0045 (9)
C9	0.0410 (11)	0.0665 (14)	0.0448 (12)	0.0226 (10)	0.0062 (9)	0.0053 (10)
C10	0.0630 (16)	0.0700 (17)	0.0566 (15)	0.0229 (13)	−0.0015 (12)	−0.0123 (12)
C11	0.0762 (17)	0.0491 (13)	0.0452 (12)	0.0237 (12)	0.0045 (11)	−0.0052 (10)
C12	0.112 (3)	0.0577 (16)	0.0650 (18)	0.0184 (17)	0.0204 (17)	0.0056 (13)
C13	0.174 (5)	0.0602 (19)	0.067 (2)	0.053 (2)	−0.006 (2)	0.0107 (15)
C14	0.144 (4)	0.094 (3)	0.085 (3)	0.075 (3)	−0.010 (3)	−0.011 (2)
C15	0.099 (3)	0.106 (3)	0.087 (2)	0.063 (2)	0.0086 (19)	0.001 (2)
C16	0.082 (2)	0.0758 (19)	0.0651 (17)	0.0404 (16)	0.0142 (14)	0.0053 (14)
C17	0.0301 (9)	0.0480 (11)	0.0444 (11)	0.0151 (8)	0.0050 (8)	−0.0017 (8)
C18	0.0417 (12)	0.0467 (12)	0.0840 (17)	0.0176 (10)	0.0137 (11)	0.0130 (12)
C19	0.0440 (13)	0.0555 (15)	0.115 (2)	0.0125 (11)	0.0274 (14)	0.0172 (15)
C20	0.0325 (11)	0.0645 (16)	0.096 (2)	0.0165 (11)	0.0112 (12)	−0.0084 (14)
C21	0.0452 (13)	0.0781 (18)	0.0735 (17)	0.0341 (13)	−0.0057 (11)	0.0041 (14)
C22	0.0416 (11)	0.0683 (15)	0.0540 (13)	0.0228 (11)	0.0021 (9)	0.0127 (11)
C23	0.0491 (13)	0.102 (2)	0.0669 (16)	0.0323 (14)	0.0147 (12)	0.0473 (16)
C24	0.0403 (12)	0.0674 (16)	0.0872 (19)	0.0164 (11)	0.0290 (12)	0.0344 (14)

### Geometric parameters (Å, º)

**Table:**

S1—O1	1.4378 (19)	C10—H10B	0.9700
S1—O2	1.4264 (18)	C10—C11	1.515 (4)
S1—C7	1.813 (2)	C11—C12	1.372 (4)
S1—C17	1.765 (2)	C11—C16	1.382 (4)
F1—C12	1.353 (5)	C12—C13	1.398 (6)
O3—C9	1.221 (3)	C13—H13	0.9300
O4—C4	1.367 (2)	C13—C14	1.385 (6)
O4—C24	1.428 (3)	C14—H14	0.9300
O5—C5	1.363 (2)	C14—C15	1.332 (6)
O5—C23	1.423 (3)	C15—H15	0.9300
N1—H1	0.8600	C15—C16	1.371 (5)
N1—C8	1.443 (3)	C16—H16	0.9300
N1—C9	1.338 (3)	C17—C18	1.386 (3)
C1—C2	1.380 (3)	C17—C22	1.377 (3)
C1—C6	1.394 (3)	C18—H18	0.9300
C1—C7	1.513 (2)	C18—C19	1.387 (3)
C2—H2	0.9300	C19—H19	0.9300
C2—C3	1.394 (3)	C19—C20	1.371 (4)
C3—H3	0.9300	C20—H20	0.9300
C3—C4	1.379 (3)	C20—C21	1.358 (4)
C4—C5	1.403 (3)	C21—H21	0.9300
C5—C6	1.383 (3)	C21—C22	1.393 (3)
C6—H6	0.9300	C22—H22	0.9300
C7—H7	0.9800	C23—H23A	0.9600
C7—C8	1.530 (3)	C23—H23B	0.9600
C8—H8A	0.9700	C23—H23C	0.9600
C8—H8B	0.9700	C24—H24A	0.9600
C9—C10	1.518 (4)	C24—H24B	0.9600
C10—H10A	0.9700	C24—H24C	0.9600
			
O1—S1—C7	107.85 (11)	C11—C10—H10B	107.9
O1—S1—C17	107.54 (11)	C12—C11—C10	124.0 (3)
O2—S1—O1	119.44 (13)	C12—C11—C16	115.0 (3)
O2—S1—C7	107.41 (11)	C16—C11—C10	121.0 (3)
O2—S1—C17	108.68 (11)	F1—C12—C11	116.5 (4)
C17—S1—C7	105.03 (9)	F1—C12—C13	120.1 (4)
C4—O4—C24	117.09 (18)	C11—C12—C13	123.3 (4)
C5—O5—C23	117.34 (16)	C12—C13—H13	121.3
C8—N1—H1	118.2	C14—C13—C12	117.4 (3)
C9—N1—H1	118.2	C14—C13—H13	121.3
C9—N1—C8	123.6 (2)	C13—C14—H14	119.4
C2—C1—C6	118.69 (18)	C15—C14—C13	121.2 (4)
C2—C1—C7	120.93 (18)	C15—C14—H14	119.4
C6—C1—C7	120.33 (17)	C14—C15—H15	120.2
C1—C2—H2	119.7	C14—C15—C16	119.6 (4)
C1—C2—C3	120.55 (19)	C16—C15—H15	120.2
C3—C2—H2	119.7	C11—C16—H16	118.3
C2—C3—H3	119.6	C15—C16—C11	123.5 (3)
C4—C3—C2	120.83 (19)	C15—C16—H16	118.3
C4—C3—H3	119.6	C18—C17—S1	118.95 (18)
O4—C4—C3	125.98 (18)	C22—C17—S1	120.22 (17)
O4—C4—C5	115.10 (18)	C22—C17—C18	120.8 (2)
C3—C4—C5	118.92 (18)	C17—C18—H18	120.5
O5—C5—C4	115.25 (17)	C17—C18—C19	119.0 (2)
O5—C5—C6	124.92 (18)	C19—C18—H18	120.5
C6—C5—C4	119.83 (18)	C18—C19—H19	120.0
C1—C6—H6	119.4	C20—C19—C18	120.1 (3)
C5—C6—C1	121.17 (18)	C20—C19—H19	120.0
C5—C6—H6	119.4	C19—C20—H20	119.6
S1—C7—H7	107.1	C21—C20—C19	120.7 (2)
C1—C7—S1	107.66 (13)	C21—C20—H20	119.6
C1—C7—H7	107.1	C20—C21—H21	119.8
C1—C7—C8	116.05 (16)	C20—C21—C22	120.5 (2)
C8—C7—S1	111.53 (14)	C22—C21—H21	119.8
C8—C7—H7	107.1	C17—C22—C21	118.9 (2)
N1—C8—C7	111.25 (18)	C17—C22—H22	120.5
N1—C8—H8A	109.4	C21—C22—H22	120.5
N1—C8—H8B	109.4	O5—C23—H23A	109.5
C7—C8—H8A	109.4	O5—C23—H23B	109.5
C7—C8—H8B	109.4	O5—C23—H23C	109.5
H8A—C8—H8B	108.0	H23A—C23—H23B	109.5
O3—C9—N1	123.5 (2)	H23A—C23—H23C	109.5
O3—C9—C10	120.3 (2)	H23B—C23—H23C	109.5
N1—C9—C10	116.2 (2)	O4—C24—H24A	109.5
C9—C10—H10A	107.9	O4—C24—H24B	109.5
C9—C10—H10B	107.9	O4—C24—H24C	109.5
H10A—C10—H10B	107.2	H24A—C24—H24B	109.5
C11—C10—C9	117.8 (2)	H24A—C24—H24C	109.5
C11—C10—H10A	107.9	H24B—C24—H24C	109.5

### Hydrogen-bond geometry (Å, º)

**Table:**

*D*—H···*A*	*D*—H	H···*A*	*D*···*A*	*D*—H···*A*
N1—H1···O4^i^	0.86	2.46	3.207 (2)	145
N1—H1···O5^i^	0.86	2.50	2.945 (3)	113

Symmetry code: (i) *x*−1, *y*, *z*.
